# Radical Hysterectomy with Pelvic Lymphadenectomy: Indications, Technique, and Complications

**DOI:** 10.1155/2010/587610

**Published:** 2010-09-01

**Authors:** Rachel A. Ware, John R. van Nagell

**Affiliations:** Division of Gynecologic Oncology, Department of Obstetrics and Gynecology, The University of Kentucky Chandler Medical Center and Markey Cancer Center, 800 Rose Street, Lexington, KY 40536-0293, USA

## Abstract

Radical hysterectomy with pelvic lymphadenectomy remains the treatment of choice for women with Stages IA_2_ and IB_1_ carcinoma of the cervix, and selected patients with Stage II endometrial cancer. Improvement in surgical techniqe, administration of prophylactic antibiotics, thromboemolic prophylaxis, and advances in critical care medicine have resulted in lower operative morbidity associated with this procedure. Major urinary tract complications such as ureteral injury or vesico-vaginal fistula are now extremely rare (<1%). Five-year survival rates following this procedure vary according to a number of clinical and histologic variables, and may be as high as 90% in women without lymph node metastases.

## 1. Introduction

The number of patients with early stage cervical cancer has steadily increased with the widespread use of the Papanicolaou test for screening. In 2009, it is estimated that there will be 11,070 new cases of cervical cancer in the United States and nearly 500,000 new cases worldwide. Approximately 85% of newly diagnosed cervical cancer in industrialized countries are expected to have localized or regional disease [1, 2]. With the trend toward early detection, more patients with invasive cervical cancer are diagnosed with early stage disease and are candidates for primary surgical treatment with radical hysterectomy and pelvic lymphadenectomy.

In 1898, Ernst Wertheim of Vienna described the operation of radical hysterectomy including removal of the parametrium and pelvic lymph nodes. In 1905, Wertheim reported outcomes of the first 270 patients treated by radical hysterectomy, which included an operative mortality rate of 18% and a major morbidity rate of 31%. Since that time, radical hysterectomy with pelvic lymphadenectomy has been performed with modifications in surgical technique as the major surgical treatment for early stage invasive cervical cancer [3, 4]. The use of prophylactic antibiotics, thromboembolic prophylaxis, administration of blood products, and advances in postoperative and critical care medicine all have lowered operative morbidity, and increased the survival rate of cervical cancer patients treated with this operation.

## 2. Indications

The primary indication for radical hysterectomy with pelvic lymphadenectomy is Stage I invasive cervical cancer. Early invasive cervical cancer is divided by the International Federation of Gynecology and Obstetrics (FIGO) Staging System into Stage IA_1_, which includes lesions invading the cervical stroma to a depth of 3 mm, or less and a maximum horizontal spread of 7 mm, and Stage IA_2_, which includes lesions with stromal invasion of 3–5 mm and a maximum horizontal spread of 7 mm [5]. These diagnoses can be made only after careful histologic evaluation of a conization specimen using an ocular micrometer to establish the depth of stromal invasion. Patients with Stage IB_1_ cervical cancer have microscopic evidence of stromal invasion >5 mm, horizontal spread >7 mm or a clinically visible cervical lesion ≤4.0 cm diameter. In patients with FIGO Stage IA_1_ cervical cancer and no evidence of lymph vascular space invasion (LVSI), conservative therapy with cervical conization or simple hysterectomy is appropriate [6–11]. However, patients with Stage IA_2_ or Stage IB_1_ cervical cancer have a significant risk of lymph nodal spread and should be treated by radical hysterectomy and pelvic lymphadenectomy. Patients with Stage IB_2_ or Stage IIA cervical cancer are treated with chemoradiation or combined therapy in many institutions. However, selected patients with Stage IB_2_ or IIA cervical cancer may be treated with radical hysterectomy and pelvic lymphadenectomy [12–14]. 

Radical hysterectomy may also be considered in the treatment recurrent cervical cancer. This procedure is appropriate only in patients with small central recurrences, following primary radiation of early stage disease. Maneo and colleagues, for example reported that radical hysterectomy is a safe alternative to pelvic exenteration in patients with Stage IB/IIA cervical cancer treated by primary radiation therapy, who have a recurrence <4 cm in diameter without evidence of ureteral obstruction or parametrial involvement [15].

Finally, radical hysterectomy with pelvic lymphadenectomy is indicated in patients with endometrial cancer and endocervical involvement (FIGO Stage II disease). Boente reviewed the clinical, surgical, and histopathologic data from 202 patients with endometrial adenocarcinoma and cervical involvement, and reported a survival advantage for patients treated by radical hysterectomy with pelvic lymphadenectomy when compared to total abdominal hysterectomy. This advantage was most notable in patients with multiple high-risk factors [16]. Radical hysterectomy with pelvic lymphadenectomy alone can be therapeutic in selected patients with Stage II endometrial cancer, thereby avoiding the morbidity associated with combination therapy [17].

## 3. Preoperative Evaluation

Prior to undergoing radical hysterectomy, patients should have a thorough evaluation to insure that there are no major medical contraindications to surgery. The anesthesiologist should be aware of the potential for blood loss in patients undergoing this procedure and should make preparation for central venous access as well as the availability of properly typed and cross-matched blood. A prophylactic antibiotic, usually a first generation cephalosporin, is given within 30 minutes of skin incision [18]. Heparin 5000 units is given subcutaneously prior to surgery and three times daily in the postoperative period for thromboembolic prophylaxis. In addition, sequential compression devices (SCDs) are placed on both lower extremities immediately prior to surgery, and are left in place until the patient is ambulating [19].

## 4. Surgical Technique

**Figure 1:**
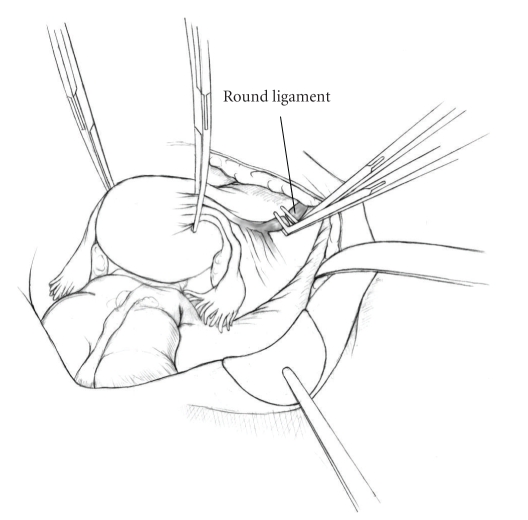


**Figure 2:**
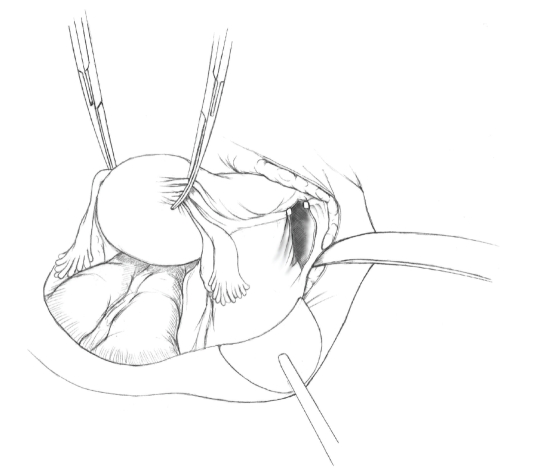


**Figure 3:**
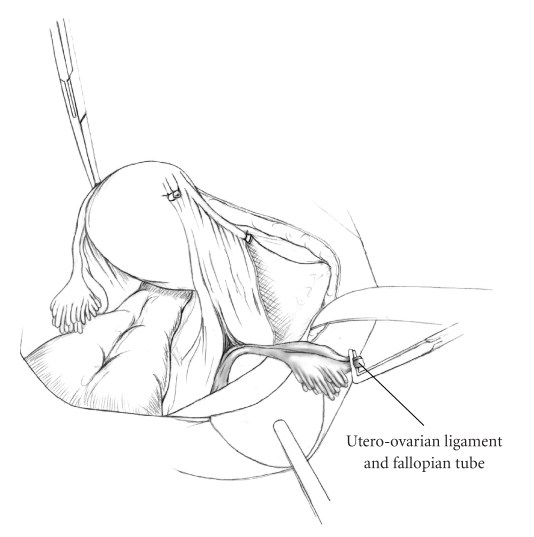


**Figure 4:**
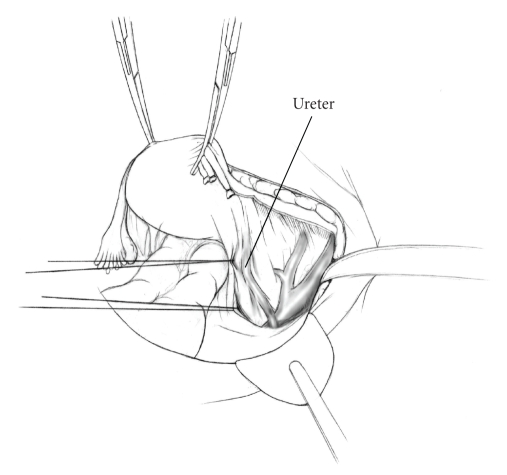


**Figure 5:**
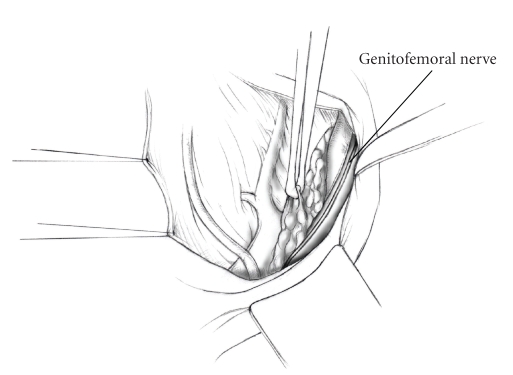


**Figure 6:**
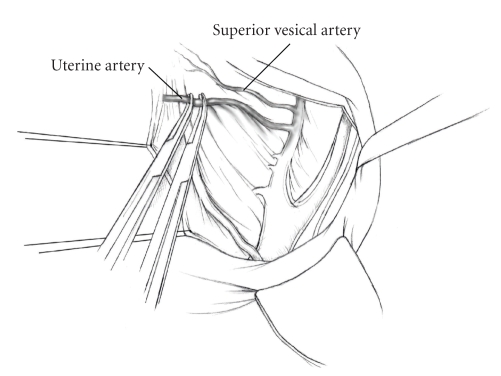


**Figure 7:**
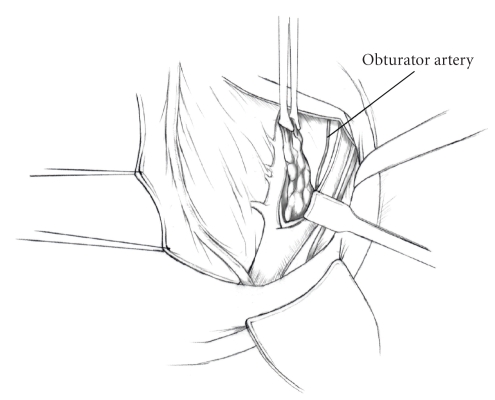


**Figure 8:**
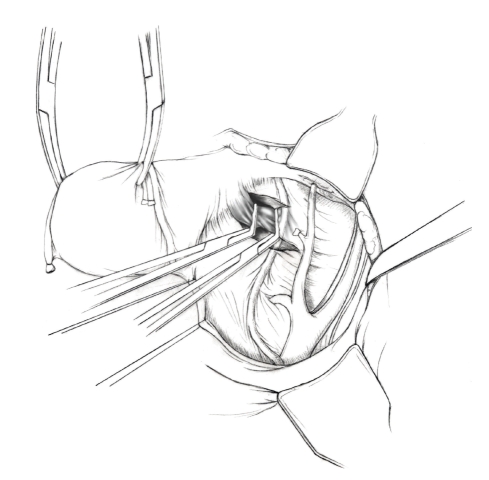


**Figure 9:**
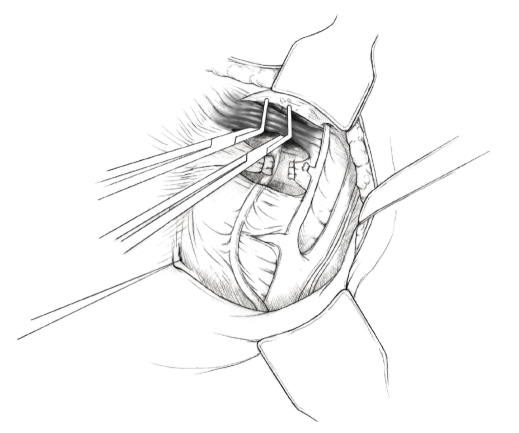


**Figure 10:**
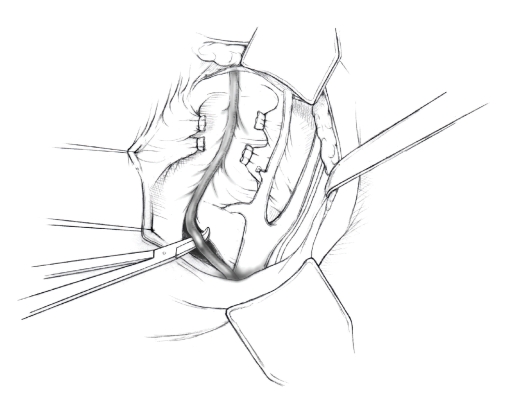


**Figure 11:**
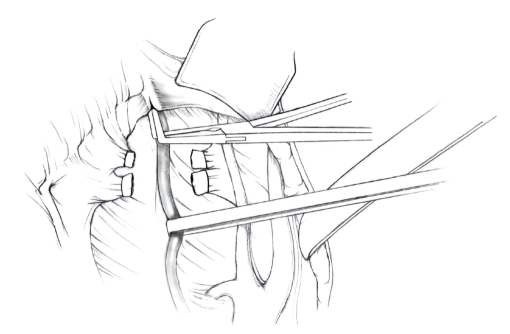


**Figure 12:**
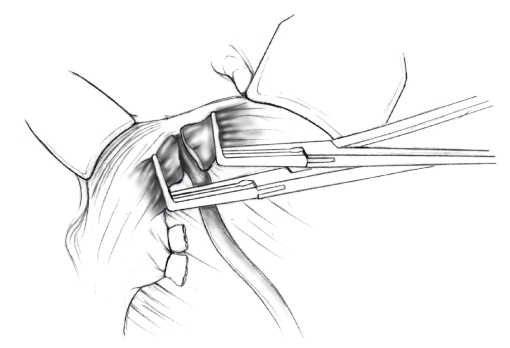


**Figure 13:**
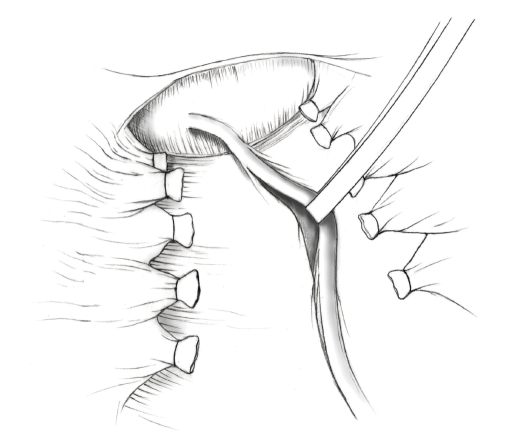


**Figure 14:**
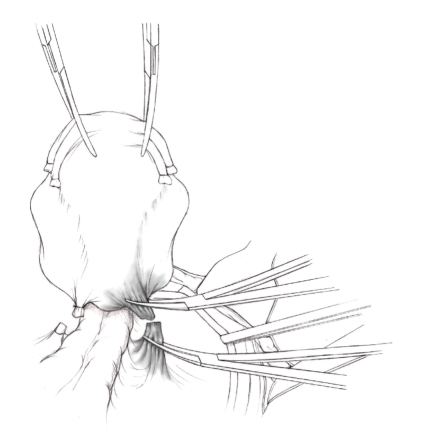


**Figure 15:**
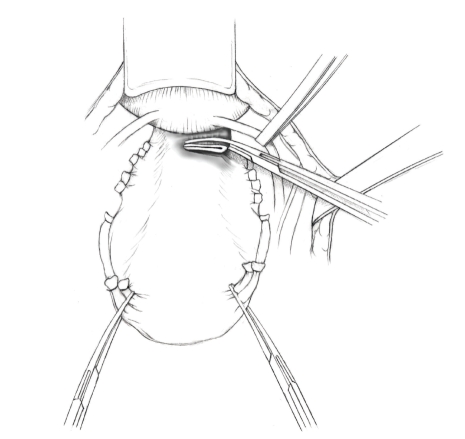


**Figure 16:**
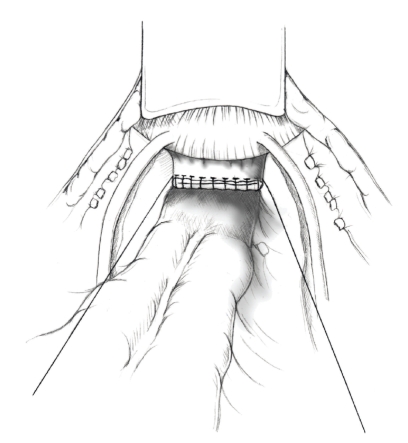


1. The patient is placed in supine position and the abdomen and vagina are prepped. A foley catheter is placed in the patient's bladder, and SCD's are placed on both lower extremities.
2. A vertical midline incision is made 3 cm above the umbilicus and is extended inferiorly to the pubic symphysis. A Bookwalter retractor is placed, and right-angle or body wall retractors are used to retract the pelvic sidewalls.
3. Prior to initiating the pelvic procedure, the entire abdominal cavity is evaluated for evidence of metastatic disease. This includes all surfaces of the liver and diaphragm, the celiac plexus, omentum, small, and large bowel surfaces as well as the mesentery. Pelvic and para-aortic lymph nodes are palpated, and any enlarged or suspicious nodes are excised and sent for histologic evaluation.
4. The bowel is packed into the upper abdomen using warm, moist laparotomy sponges. Two 8-inch Kelly Clamps are placed on the uterine cornua for retraction.
5. The right round ligament is clamped, cut, and ligated at the right lateral pelvic wall (Figure 1). The anterior leaf of the right broad ligament is incised inferiorly along the lateral pelvic wall for a distance of approximately 3 cm.
6. The posterior leaf of the right broad ligament is incised superiorly along the lateral pelvic wall to the level of the infundibulopelvic ligament (Figure 2).
7. If the right ovary is to be preserved, the posterior leaf of the right broad ligament is further incised parallel and inferior to the infundibulopelvic and utero-ovarian ligaments. The right utero-ovarian ligament is then clamped, cut, and ligated, and the ovary is placed in the right iliac fossa (Figure 3).
8. If the right ovary is to be excised, the right infundibulopelvic ligament is clamped, cut, and doubly ligated at the lateral pelvic wall. The right utero-ovarian ligament is then clamped, cut, and suture ligated, and the right tube and ovary are removed.
9. Steps  (5)–(8) are then repeated on the left side.
10. The right retroperitoneal space is entered along the lateral pelvic wall, thereby exposing the common iliac, external iliac, and internal iliac arteries and associated lymph nodal tissue.
11. The ureter is identified, and two silk sutures are placed in the adjacent medial peritoneum, thereby pulling the ureter medially away from the iliac vessels (Figure 4).
12. The lymph node dissection is begun by sharply excising all lymph nodal tissue surrounding the right common iliac, external iliac, and internal iliac arteries. The lateral extent of the pelvic lymph node dissection is defined by the genitofemoral nerve (Figure 5). The external iliac and common iliac arteries are retracted laterally, and lymph nodal tissue surrounding the common iliac, external iliac, and internal iliac veins is removed by sharp dissection. Lymph nodal tissue from each of the major anatomic sites (i.e., common iliac, external iliac, internal iliac) is placed in separate containers and submitted for histologic analysis.
13. The anterior division of the internal iliac artery is identified, and the uterine artery is isolated, ligated with 2-0 silk ties, and transected. The superior vesical artery is preserved (Figure 6).
14. A vein retractor is placed on the medial aspect of the external iliac artery and vein, and the obturator nerve is identified. All lymph nodal tissue is removed from the obturator fossa by sharp dissection (Figure 7), placed in a separate container and submitted for histologic analysis.
15. Steps  (10)–(14) are repeated on the left side.
16. The right pararectal space and paravesical spaces are defined by blunt dissection, and the lateral aspect of the cardinal ligament containing the vascular web is clamped, cut, and ligated with 2-0 silk ties (Figures 8 and 9). Ligation of the left vascular web is completed in the same fashion.
17. The right ureter is dissected from the medial peritoneum at the level of the uterosacral ligament (Figure 10), and a 3/8 inch Penrose drain is placed around the ureter (Figure 11). The ureter is dissected laterally from the parametrial tunnel using right angle clamps (Figure 12). The parametrial vasculature is ligated, and the ureter is rolled laterally out of the tunnel. The right ureter is dissected free from surrounding tissue until its entrance into the bladder (Figure 13). The left ureter is then dissected free from the left parametrium in the same fashion.
18. The bladder is sharply dissected from the anterior vagina, and the peritoneum between the uterus and the rectum is incised. The anterior rectal wall is reflected away from the posterior vagina.
19. The uterus is elevated and the uterosacral ligaments are clamped, cut, and tied (Figure 14). The anterior, posterior, and lateral attachments of the uterus and parametria have now been ligated. The paravaginal tissue at the inferior margin of the dissection is clamped, cut, and tied using curved Lainz clamps (Figure 15).
20. The vagina is transected approximately 3 cm below the cervix and isolated bleeding sites on the vaginal cuff are ligated using 2-0 vicryl suture. The vagina is closed using a continuous interlocking 0 vicryl suture (Figure 16).
21. Closed suction drains may be placed in both retroperitoneal spaces at the discretion of the surgeon. These drains are brought out through the anterior abdominal wall in each lower quadrant and are sutured to the skin using 2-0 silk suture.
22. If the ovaries are retained, they are suspended to the lateral pelvic wall with 2-0 prolene, and titanium clips are placed on the suture site for future identification.
23. The abdomen is then closed in layers, using continuous 0 looped PDS in a modified Smead-Jones technique.

## 5. Complications

Complications of radical hysterectomy with pelvic lymphadenectomy are summarized in Table 1. Bladder dysfunction and lymphocyst formation are among the most common complications of radical hysterectomy and occur in 5%–15% of cases in recent reports. Bladder dysfunction results from extensive dissection of the ureters at the bladder base and transection of the uterosacral ligaments, which interrupts autonomic nerve supply to the bladder. In general, more radical dissection results in a higher frequency of bladder dysfunction. However, preservation of the superior vesical artery and blood supply to the distal ureter has resulted in a marked decrease in the frequency of vesicovaginal and ureterovaginal fistula following radical hysterectomy.

 Lymphocyst formation after radical hysterectomy and lymphadenectomy is due to interruption of efferent pelvic lymphatics and can result in lymphedema, pelvic discomfort, and infection as well as an increase in the frequency of deep venous thrombosis and pulmonary embolism. Variation in the incidence of lymphocyst formation depends on the extent of lymphadenectomy, retroperitoneal drain placement, and differences in the surgical technique used for ligating lymphatic channels. Lymphocysts can be managed with guided-percutaneous drainage or laparoscopic surgical resection [35, 36].

 The incidence of thromboembolic disease after radical hysterectomy has decreased over time, as a result of widespread implementation of thromboprophylaxis with preoperative and postoperative Heparin and lower extremity sequential compression devices. Nevertheless, it remains the leading cause of mortality in the immediate postoperative period. Multiple clinical trials have provided irrefutable evidence that thromboprophylaxis decreases the risk of deep venous thrombosis and pulmonary embolus. Pulmonary embolus has been cited as the most common cause of preventable hospital death, therefore making thromboprophylaxis the number one strategy to improve safety for patients undergoing major pelvic surgery [19].

## 6. Survival

The 5-year survival in patients with early stage cervical cancer treated with radical hysterectomy and pelvic lymphadenectomy varies between 80% and 95% according to a number of clinical and histologic findings, and is summarized in Table 2. Patients with low-risk early-stage disease, undergoing radical surgical treatment have a survival of nearly 100% [20]. However, patients with more advanced disease have lower reported outcomes. Several risk factors related to poor prognosis include large tumor volume, deep stromal invasion, presence of lymph vascular space invasion, and lymph node metastases [31–33]. A thorough analysis of these factors is helpful in determining which patients may benefit from postoperative therapy following radical hysterectomy.

## 7. Summary

Radical hysterectomy with pelvic lymphadenectomy is the treatment of choice for healthy women with stage IA_2_-IB_1_ cervical carcinoma. Women with nonbulky IB_2_ and IIA cervical carcinoma, centrally recurrent disease, and endometrial carcinoma with cervical involvement may also be considered for surgical treatment by radical hysterectomy. Improvements in surgical technique, prophylactic antibiotics, thromboembolic prophylaxis, administration of blood products, and advances in postoperative and critical care medicine all have lowered operative morbidity from this procedure. Five-year survival rates in excess of 90% can be achieved when this procedure is performed for the proper indications.

## Figures and Tables

**Table 1:** Complications of Radical Hysterectomy.

Author	# of Pts	Wound Infection (%)	Ureteral Injury (%)	Bladder Injury (%)	Blood Vessel Injury (%)	Urinary Tract Fistula (%)	Bladder Dysfunction (%)	Pulmonary Embolism or DVT (%)	Lympho-cysts (%)	Intestinal Obstruction (%)
Cai et al. 2009 [20]	480	0.63	0.83	0.83	0.21	1.0	12.9	—	5.8	—
Manchana et al. 2009 [21]	290	—	—	—	—	—	14.5	—	9.3	—
Likic et al. 2008 [22]	529	—	1.32	—	—	—	—	—	—	—
Pikaart et al. 2007 [23]	201	4.0	1.5	—	—	0.5	7.5	1.5	1.0	—
Wu et al. 2006 [24]	219	—	—	—	—	9.5	19.9	—	7.8	—
Landoni et al. 2001 [13]	109	—	0.92	—	—	0.92	14.7	2.8	11.9	—
Zorlu et al. 1998 [25]	115	10.4	0.87	2.6	8.7	—	—	—	—	—
Abr$\tilde{\text{a}}$o et al. 1997 [26]	302	—	—	—	—	2.9	9.2	—	—	—
Finan et al. 1996 [27]	229	—	—	—	—	—	—	4.4	—	—
Magrina et al. 1995 [28]	375	—	—	—	—	0.3	—	0.8	0.8	1.3
Benedetti-Panici et al. 1993 [29]	84	—	—	—	—	7.1	—	4.8	21.4	—
Ayhan et al. 1991 [30]	270	—	—	—	—	—	16.2	—	6.4	—

**Table 2:** Five-year survival following radical hysterectomy for cervical cancer.

Author	# of Pts	Stage	Size of Lesion (cm)	Depth of Stromal Invasion	Lymphvascular Space Invasion	Lymph Node Status	5-Year Survival (%)	*P* value
Sartori et al. 2007 [31]	454	IB-IIA	—	—	—	All	80	<.01
						Negative	88	
						Positive	57	
Ho et al. 2004 [32]	197	IB-II	—	All	—	—	82	
				Inner 2/3	—	—	88.8	.003
				Outer 1/3	—	—	73.8	
					Negative	—	89.5	<.001
					Positive	—	68.4	
						Negative	87.3	<.001
						Positive	67.2	
Ayhan et al. 2004 [33]	393	IB	All	—	—	—	91	
			≤4	—	—	—	91.0	.012
			>4	—	—	—	85.9	
				—	Negative	—	93.7	.009
				—	Positive	—	86.0	
Landoni et al. 2001 [13]	238	IB-IIA	All	—	—	—	79	
								
Tsai et al. 1999 [12]	222	IB-IIA	All	—	—	—	76	
			≤4	—	—	—	88	.0003
			>4	—	—	—	67	
						Negative	87	.0011
						Positive	71	
Lai et al. 1999 [34]	827	IB-II	<2	—	—	—	90.6	.0001
			2–4	—	—	—	82.7	
			>4	—	—	—	69.1	
				Inner 2/3	—	—	90.3	.0001
				Outer 1/3	—	—	74.9	
					Negative	—	86.3	.0002
					Positive	—	76.5	
						Negative	87.3	.0001
						Positive	68.2	
